# Risk perception and trust in the relationship between knowledge and HPV vaccine hesitancy among female university students in China: a cross-sectional study

**DOI:** 10.1186/s12889-024-18166-w

**Published:** 2024-03-01

**Authors:** Xing Chen, Lei Wang, Yan Huang, Luying Zhang

**Affiliations:** 1 School of Public Health, Fudan University, Shanghai, PR China; 2 School of Public Administration and Emergency Management, Institute of Public Policy, Jinan University, Guangzhou, PR China; 3 Center for Chinese Public Administration Research, School of Government, Sun Yat-sen University, Guangzhou, PR China

**Keywords:** HPV, Vaccine hesitancy, Mediating role, Knowledge, University students

## Abstract

**Background:**

Cervical cancer poses a heavy health burden in China, with the second highest incidence and mortality rate among female tumors, yet human papillomavirus (HPV) vaccination rate among female university students remain remains low. This study conducted a cross-sectional survey to assess the degree of HPV vaccine hesitancy among female university students and to explore the potential association between knowledge, risk perception, trust, and HPV vaccine hesitancy.

**Methods:**

A total of 1,438 female university students from four Chinese cities were recruited through stratified, multistage, cluster sampling method. The mediation model was constructed using the Bootstrap method, introducing trust and risk perception as mediating variables to examine the effect of knowledge on HPV vaccine hesitancy.

**Results:**

The study found that 8.9% (95%CI:7.4%∼10.4%) of the female university students exhibited HPV vaccine hesitancy. Pearson’s correlation analysis revealed a negative association between vaccine hesitancy and knowledge, risk perception, and trust. The mediation model showed that knowledge had significant indirect effects on HPV vaccine hesitancy through trust (indirect effect: -0.224, 95% CI: -0.293 ∼ -0.167) and risk perception (indirect effect: -0.013, 95% CI: -0.033 ∼ -0.002).

**Conclusion:**

HPV vaccine hesitancy among female university students has mitigated, but still needs to be addressed. In addition, trust and risk perception are mediators mediating the relationship between knowledge with HPV vaccine hesitancy. Therefore, there is a need to strengthen public health education to improve knowledge, with a particular focus on providing information about trust and risk perception to reduce HPV vaccine hesitancy.

## Introduction

Cervical cancer, caused primarily by persistent human papillomavirus (HPV) infection, is one of the leading causes of cancer deaths among women in developing countries [1–2]. Cervical cancer in China has the second highest incidence rate (17.69/10^5^) and mortality rate (5.52/10^5^) of female tumors, and is one of the countries with a large burden of cervical cancer [3–4].

The HPV vaccine is effective in preventing HPV infection by stimulating the body to produce antibodies that bind to the virus [5]. Studies have shown that 5–8 years after vaccination, HPV 16 and 18 infections were significantly reduced by 83% in girls aged 13–19 years and by 66% in women aged 20–24 years [6]. Therefore, promoting timely HPV vaccination for age-appropriate women will contribute to the early realization of the goal of eliminating cervical cancer. However, the vaccination rate among female university students in China is lower than 15% [7–9].

In the face of low vaccination rates, the WHO Strategic Advisory Group of Experts noted that vaccine hesitancy is a growing challenge for immunization programs [10]. Vaccine hesitancy refers to delay or refusal of vaccination despite the availability of vaccination services [11–12]. It has become the primary reason for the low or declining HPV vaccination rate in many countries recently, including Japan, Brazil and India [13–16]. In China, the HPV vaccine hesitancy rate among female university students was reported as 37.57% [17]. Therefore, more efforts are still needed to reduce HPV vaccine hesitancy among female university students in China.

Numerous studies have found that knowledge of the HPV and HPV vaccine significantly affects HPV vaccine hesitancy [18–19]. Studies have found that increased HPV knowledge for the public can help improve vaccination rates [20]. Studies have also linked risk perception of HPV and trust in HPV vaccine to knowledge and HPV vaccine hesitancy. Studies indicate that high levels of knowledge were associated with greater risk perception of HPV, and the latter was associated with HPV vaccine hesitancy [21–22]. Meanwhile, mistrust in HPV vaccine was consistently associated with HPV vaccine hesitancy and lower HPV vaccine uptake, so increasing public trust could help alleviate HPV vaccine hesitancy [23–25].

**Fig. 1:**
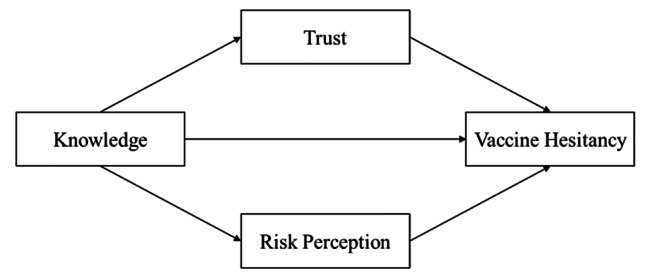
The hypothetical conceptual model

So far, little was known about the role and possible pathways of risk perception of HPV and trust in HPV vaccine. Only a few studies have found a mediating role for the above factors in the Covid-19 vaccine [26–27]. Therefore, this study aimed to explore the role of trust and risk perceptions between knowledge and HPV vaccine hesitancy among female university students across China. To achieve this, a hypothetical model was developed based on the widely used Knowledge, Attitude, and Practice (KAP) model in vaccination and vaccine hesitancy research (see Fig. 1) [28–29]. According to our knowledge, this study is the first to explore the role of trust and risk perceptions in the relationship between knowledge and HPV vaccine hesitancy among female university students in China.

## Materials and methods

### Study setting

To date, HPV vaccine has not yet been included in national immunization plan in China. However, China has been actively working towards eliminating cervical cancer. The Chinese government has released the Action Plan for Accelerating the Elimination of Cervical Cancer (2023–2030), which involves schools in organizing and mobilizing efforts to increase the willingness of eligible girls to receive HPV vaccines. Some cities already initiated free HPV vaccine policies for junior middle school girls.

### Study design and population

This study conducted a cross-sectional survey using stratified, multistage, cluster sampling method. First, we selected four representative cities in China based on their economic level and geographical location. Among them, Shanghai is a municipality directly under the central government, and Guangzhou, Wuhan, and Nanning are the capital cities of the eastern, central, and western regions, respectively. Second, according to the categories of universities, 3 or 4 key universities, general universities, and specialized colleges were selected as sample universities in each city. Finally, one college or class in each sample university was randomly selected to distribute the questionnaire. In this study, female students from a total of 56 universities participated in the survey. Inclusion criteria of the sample population were (1) voluntary participation in this study and written informed consent was obtained; (2) junior college, undergraduate, and postgraduate students enrolled in the sample universities; and (3) females aged 18 years or older. The exclusion criteria were (1) the questionnaire took less than 120 s to fill out; (2) the IP address where the questionnaire was filled out did not match the IP address where the school was located; and (3) the questionnaire was not completed. This study was ethically reviewed and approved by the Institutional Review Board, School of Public Health, Fudan University (IRB#2022-08-0992).

### Sample size

The sample size was estimated using the calculation formula: $n=\frac{{Z}_{1-\alpha /2}^{2}\times p(1-p)}{{\delta }^{2}}$(α = 0.05, *Z*_1*−α*/2_ = 1.96). In this study, *n* represents the number of female university students to be surveyed and *p* indicates the expected HPV vaccine uptake intention of female university students. According to two recent meta-analyses from China, the uptake intention of HPV vaccine among Chinese female university students was 68.0-71.8% [30–31]. Therefore, this study assumed that the HPV vaccine uptake intention of female university students in China was 70.0%. Substituting *p* = 0.70 into the above calculation formula, we can calculate that n equals 322. This means that 1288 female university students would be surveyed in four cities. Considering the uncontrollable factors in the study, the sample size was appropriately enlarged by 10% to 1417 in this study. Furthermore, we utilized the data collected from the pre-survey to determine the appropriate sample size for the mediation analysis. This was done by employing the Monte Carlo calculation method proposed by Schoemann [32]. The calculations revealed that a sample size greater than 1400 would result in a conventional power exceeding 0.8.

### Measures

#### HPV vaccine hesitancy

Firstly, participants were asked the question of “Have you already received or made an appointment for cervical cancer (HPV) vaccine?“. The answers were vaccinated (at least 1 vaccination), appointed (not yet injected), and not yet vaccinated. We defined participants who have received or made an appointment for HPV vaccine as without HPV vaccine hesitancy, based on the WHO definition of vaccine hesitancy. Accordingly, their vaccine hesitancy score was assigned a value of 0. If participants did not receive or make an appointment for HPV vaccination, they would be further asked “To what extent are you hesitant to get cervical cancer (HPV) vaccine?“. The answers were a score of 0–10. Then, we classified those who scored 0 as the no hesitation group, those who scored 1–9 as the hesitation group, and those who scored 10 as the refusal to vaccinate group.

#### Vaccine knowledge

Participants’ knowledge of the HPV and HPV vaccine was measured through four questions: “Do you know about HPV”, “Do you know the main ways of transmission of HPV”, “Have you heard of the HPV vaccine”, and “The best time to get your first HPV vaccination”. The answers to all questions were multiple choice, with only one option being correct. Each question was worth 1 point, all the responses to these four items were summed up to create one score. The possible score ranged from 0 to 4.

#### Vaccine trust

Trust in HPV vaccine was assessed using five items: “Your trust in the technical ability of domestic HPV vaccine production and related companies”, “Your trust in the technical ability of imported HPV vaccine production and related companies”, “Your belief that the HPV vaccine can prevent cervical cancer and other diseases”, “Your belief that vaccination against HPV is necessary”, and “Your trust in medical professionals’ recommendations for HPV vaccination”. A 5-point Likert scale was used, ranging from " strongly disagree " to " strongly agree “. The total score of the five items is used as the trust score, and the possible score ranges from 5 to 25.

#### Risk perception

To assess risk perceptions of HPV, three items were used, including “How dangerous do you think cervical cancer is to your health”, “How likely do you think it is to develop cervical cancer”, and “How fearful do you feel about cervical cancer”. An 11-point Likert scale was used, ranging from “not at all serious” to “very serious”. The total score of the three items is used as the risk perception score, and the possible score ranges from 0 to 30.

### Data collection

The data were collected from June to July 2022. Using the preliminary questionnaire designed by the research team, 60 female college students were randomly selected in a university for a pre-survey, and the problems found in the pre-survey were corrected to form the official online questionnaire. The online questionnaire was sent to female university students through a link or QR code published by the teacher to the class. The female university students participated in the survey by clicking on the web link or scanning the QR code of Wenjuanxing (an online survey tool) with their cell phones or computer. Sociodemographic issues include age, city, education level, major, residence, medical insurance, living expenses, etc. After the questionnaires were collected, the quality of the questionnaires was checked by members of the research team. Quality control criteria: (1) The questionnaire response time was greater than 120 s, (2) The city of study or IP address was the same as the city where the university was located, (3) The questionnaire content was answered completely. Finally, a total of 1,498 online questionnaires were collected in this study, of which 1,438 were valid through quality check.

### Statistical analysis

Data analysis was performed using IBM SPSS and SPSS AMOS (IBM Corp., Armonk, NY, USA). The chi-square test and one-way ANOVA were used to test the differences in HPV vaccine hesitancy among female university students with different socio-demographic characteristics. Pearson correlation analysis was used to test the correlation between HPV vaccine hesitancy, knowledge, trust, and risk perception. Structural equation modeling was used to explore the mediating effects of trust and risk perception between knowledge and vaccine hesitancy. The control variables are education, family history of cancer, and self-pay vaccination history. All variables in SEM were observed, identification was established using covariance algebra, parameter estimation was performed using maximum likelihood, and model fit was verified using χ2/df, GFI, AGFI, RMSEA. Mediation effects analysis was performed using bootstrap analysis with 5000 bootstrap samples and 95% bias-corrected confidence intervals. For all analyses, *p*-values of < 0.05 was considered statistically significant.

## Results

### Demographic characteristics of HPV vaccine hesitancy

In the sample population, 30.6% were from Shanghai, 24.3% were from Wuhan, 22.9% were from Guangzhou, and 22.3% were from Nanning. The percentages of junior college, undergraduate, and Postgraduate students were 17.0%, 67.5%, and 15.5%, respectively. The main majors of the respondents were social sciences and medicine, with 43.7% and 45.8% respectively. Most students had no family history of cancer (91.2%) and self-paying vaccinations (75.6%), lived in rural areas (52.9%) and had health insurance (93.9%). 63.8% of female university students had monthly living expenses from 1000 to 2000 CNY (equals to USD 143–286). The vaccine hesitancy group scored 2.95 for knowledge, 19.20 for risk perception, and 20.58 for trust, all lower than the no hesitancy group. See Table 1.

**Table 1:** Demographic characteristics of female university students by HPV vaccine hesitancy

Variables		*N*	The Attitude to HPV Vaccination, *n* (%)			*P*
			No Hesitancy	Hesitancy	Refusal	
Age		1438(100.00)	21.06 ± 2.30	20.78 ± 2.52	21.80 ± 2.78	0.147
City						
	Shanghai	440(30.60)	392(89.09)	43(9.77)	5(1.14)	0.338
	Wuhan	349(24.27)	318(91.12)	25(7.16)	6(1.72)	
	Guangzhou	329(22.88)	301(91.49)	26(7.90)	2(0.61)	
	Nanning	320(22.25)	279(87.19)	34(10.63)	7(2.19)	
Education						
	Junior college	245(17.04)	215(87.76)	28(11.43)	2(0.82)	0.011
	Undergraduate	970(67.45)	864(89.07)	93(9.59)	13(1.34)	
	Postgraduate	223(15.51)	211(94.62)	7(3.14)	5(2.24)	
Major						
	Technology and Agriculture	151(10.50)	138(91.39)	11(7.28)	2(1.32)	0.885
	Social sciences	628(43.67)	558(88.85)	61(9.71)	9(1.43)	
	Medicine	659(45.83)	594(90.14)	56(8.50)	9(1.37)	
Residence						
	Rural	761(52.92)	675(88.70)	74(9.72)	12(1.58)	0.404
	Urban	677(47.08)	615(90.84)	54(7.98)	8(1.18)	
Medical insurance						
	Yes	1350(93.88)	1211(89.70)	120(8.89)	19(1.41)	0.977
	No	88(6.12)	79(89.77)	8(9.09)	1(1.14)	
Living expense (CNY)						
	<1000	177(12.31)	150(84.75)	22(12.43)	5(2.82)	0.156
	1001–2000	918(63.84)	830(90.41)	78(8.50)	10(1.09)	
	>2000	343(23.85)	310(90.38)	28(8.16)	5(1.46)	
Family history of cancer						
	Yes	127(8.83)	116(91.34)	4(3.15)	7(5.51)	<0.001
	No	1311(91.17)	1174(89.55)	124(9.46)	13(0.99)	
Self-pay vaccination history						
	Yes	351(24.41)	328(93.45)	20(5.70)	3(0.85)	0.030
	No	1087(75.59)	962(88.50)	108(9.94)	17(1.56)	
Knowledge		1438(100.00)	3.35 ± 0.80	2.95 ± 1.12	2.45 ± 1.23	<0.001
Risk Perception		1438(100.00)	19.20 ± 5.58	17.42 ± 6.39	11.60 ± 5.86	<0.001
Trust		1438(100.00)	20.58 ± 2.19	17.91 ± 2.20	16.40 ± 2.68	<0.001

### HPV vaccine hesitancy

In this survey, 23.6% (339) of female university students had received the HPV vaccine, and 10.6% (153) have already made an appointment for HPV vaccination. Of the remaining female university students, 84.4% (798) expressed their willingness to obtain the HPV vaccine, 13.5% (128) were hesitant, and 2.1% (20) said they refused to get the HPV vaccine. Therefore, the hesitation rate of female university students about HPV vaccine in this survey was 8.9%. Analysis showed that HPV vaccine hesitancy among female university students was associated with education, family history of cancer, self-pay vaccination history, knowledge, risk perception, and trust (*P* < 0.05). And HPV vaccine hesitation is not related to age, city, major, residence, medicine insurance, and living expense (*P*>0.05). See Table 1.

### Bivariate correlations among primary variables

Table 2 provided the correlations among the primary variables studied. Correlation analysis showed that HPV vaccine hesitancy was negatively associated with knowledge, risk perception, and trust among female university students (*r*=-0.158, *P* < 0.01; *r*=-0.122, *P* < 0.01; *r*=-0.366, *P* < 0.01). Knowledge is positively correlated with trust and risk perception among female university students (*r* = 0.285, *P* < 0.01; *r* = 0.086, *P* < 0.01).

**Table 2:** Correlation analysis between primary variables

Variables	Knowledge	Risk Perception	Trust	Vaccine Hesitancy
Knowledge	1.000			
Risk Perception	0.086**	1.000		
Trust	0.285**	0.156**	1.000	
Vaccine Hesitancy	-0.158**	-0.122**	-0.366**	1.000

** *P*<0.001

### Mediation effect analysis

**Fig. 2:**
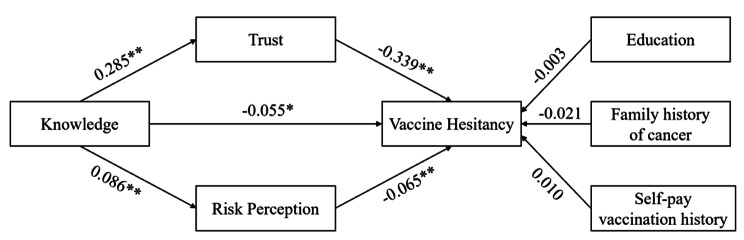
Parallel mediation model. (*P < 0.05, **P < 0.01.)

The results showed good model fit and hypothesis were valid (χ2/df = 7.744, RMSEA = 0.069(95%CI: 0.056–0.082), GFI = 0.981, AGFI = 0.955). The total mediating effect from knowledge to HPV vaccine hesitancy was statistically significant (total mediating effect: -0.364, 95% CI: -0.537 ∼ -0.207), as was the direct path from knowledge to HPV vaccine hesitancy (direct effect: -0.127). Knowledge had a significant indirect effect on HPV vaccine hesitancy through trust (indirect effect: -0.224, 95% CI: -0.293 ∼ -0.167). Also, Knowledge had a significant indirect effect on vaccine hesitancy through risk perception (indirect effect: -0.013, 95% CI: -0.033 ∼ -0.002). More details about the mediation model were demonstrated in Fig. 2; Table 3.

**Table 3:** Mediating effects of knowledge and HPV vaccine hesitancy

Path	Estimate	95%CI		*P*
		Lower	Upper	
Total effect: Knowledge → Vaccine Hesitancy	-0.364	-0.293	-0.167	<0.001
Ind1: Knowledge → Trust → Vaccine Hesitancy	-0.224	-0.293	-0.167	<0.001
Ind2: Knowledge → Risk Perception → Vaccine Hesitancy	-0.013	-0.033	-0.002	0.010

## Discussion

This study not only updated the present status of HPV vaccine hesitancy among female university students in China, but also shed light on the relationship between knowledge, trust, risk perception, and HPV vaccine hesitancy using mediation modeling. Firstly, this study found that Chinese female university students’ hesitancy to receive the HPV vaccine has decreased. Secondly, knowledge, trust, and risk perception were influential factors for HPV vaccine hesitancy. Furthermore, trust and risk perception played a mediating role on the relationship between knowledge and HPV vaccine hesitancy.

The results showed that the HPV vaccine hesitancy rate was 8.9%, which is lower than previous studies among Chinese female university students, such as Guangxi (30.4%) and Beijing (37.57%) [17, 33]. This may be because this study is a multicenter cross- sectional survey with multiple sample cities selected based on the economic level and geographic location, whereas previous studies had samples from a single city. In addition, the COVID-19 pandemic has to some extent influenced the fear of diseases and enhanced the willingness to vaccinate [34].

In this study, the level of knowledge was negatively correlated with HPV vaccine hesitancy. Consistent with previous studies, insufficient knowledge about HPV and HPV vaccine will lead to reluctance of female university students to get HPV vaccination [35]. Typically, individuals with lower levels of knowledge are more vulnerable to misinformation, which can lead to misconceptions about immunization. Therefore, more public health education on HPV and HPV for university students is needed to improve student knowledge [36]. But there are also studies that point out that providing too much knowledge can be counterproductive [37]. Therefore, progressive interventions are needed to improve knowledge.

Mediation analysis showed that knowledge can have an indirect effect on HPV vaccine hesitancy through trust and risk perception. First, trust enables people to maintain their capacity to act in a complex environment, so people will only be willing to get vaccinated if they have enough trust in the HPV vaccine [38]. However, numerous negative rumors about HPV vaccines continue to undermine trust in HPV vaccines among knowledge-poor groups, contributing to hesitation about HPV vaccination [39]. For example, a survey in Serbia found that knowledge directly influences people’s willingness to vaccinate and is mediated by trust [40]. Second, risk perception was a subjective identification of the possible risk exposure [41], the increased knowledge about HPV and the HPV vaccine would make the risk of HPV more perceptible and then increase the willingness to get vaccinated against HPV. For example, a study in the U.S. suggests that participants with lower knowledge of HPV may also have a low perceived risk of HPV, which would influence HPV vaccination behavior [42]. In general, high levels of knowledge will favorably translate into positive attitudes, i.e., elevated levels of trust and risk perception, which contribute to lower vaccine hesitancy and good behavior [43]. Therefore, more information on trust and risk perception should be provided when raising the knowledge level of female university students. For example, the capacity of vaccine production and development companies, the preventive effect and safety of the vaccine, the likelihood of HPV infection, the risk of cervical cancer, and so on.

This study provides further insights into the association between knowledge and vaccine hesitancy, highlighting the mediating roles of trust and risk perception. Vaccine hesitancy is a widespread problem, and the results of this study can provide a reference for countries when developing interventions. To further reduce HPV vaccine hesitancy, the government needs to increase publicity about cervical cancer prevention and treatment, limit the dissemination of bad information, respond to public concerns in a timely manner, and recommend that health education be incorporated into school curricula. In addition, researchers could consider ways to improve people’s trust in the vaccine and the risk perception of the disease.

To our knowledge, this is the first study to explore the role of trust and risk perception in the relationship between knowledge and HPV vaccine hesitancy among female university students in China. This study also has some limitations. First, this study only included trust and risk perception as main determinants and did not consider other factors that might be related to the knowledge. Meanwhile, the knowledge scale may have limited sensitivity due to few items and dichotomized scoring. In the future, more nuanced measures could be considered to assess conceptual and procedural knowledge. Second, this study used an online survey in which participants self-selected to participate in the study, so there may be some sampling bias. Finally, the correlations between the main variables in this study were not strong, which suggests that further research is still needed in the future.

## Conclusions

This study investigated the current status of HPV vaccine hesitancy among Chinese female university students and explored the relationship between trust and risk perception in knowledge and vaccine hesitancy. The results suggest that the degree of HPV vaccine hesitancy among female university students has moderated. In addition, trust and risk perception mediate the relationship between knowledge and HPV vaccine hesitancy. Based on these results, it is recommended that the government and school need to enhance public health education for female university students to improve the knowledge and reduce vaccine hesitancy. Moreover, while improving knowledge, it is vital to provide additional information on trust and risk perception to enhance its effectiveness.

## Data Availability

The data that support the findings of this study are available from the corresponding author upon reasonable request.
