# Correction: Risk perception and trust in the relationship between knowledge and HPV vaccine hesitancy among female university students in China: a cross-sectional study

**DOI:** 10.1186/s12889-025-25130-9

**Published:** 2025-11-12

**Authors:** Xing Chen, Lei Wang, Yan Huang, Luying Zhang

**Affiliations:** 1 School of Public Health, Fudan University, Shanghai, PR China; 2 School of Public Administration and Emergency Management, Institute of Public Policy, Jinan University, Guangzhou, PR China; 3 Center for Chinese Public Administration Research, School of Government, Sun Yat-sen University, Guangzhou, PR China

**Correction: BMC Public Health 24**,** 667 (2024)**.

**https://doi.org/10.1186/s12889-024-18166-w**

Following publication of the original article [1], the authors would like to add the following reference under the Introduction section.

Huang Y, Chen C, Wang L, et al. HPV Vaccine Hesitancy and Influencing Factors among University Students in China: A Cross-Sectional Survey Based on the 3Cs Model. Int J Environ Res Public Health. 2022;19(21):14025.

The reference is inserted in the following statement as reference 25:

Meanwhile, mistrust in HPV vaccine was consistently associated with HPV vaccine hesitancy and lower HPV vaccine uptake, so increasing public trust could help alleviate HPV vaccine hesitancy [23–25].

The original article [1] has been updated.
